# ‘THIS IS NO COUNTRY FOR OLD (WO)MEN’? AN EXAMINATION OF THE APPROACH TAKEN TO CARE HOME RESIDENTS DURING THE COVID-19 PANDEMIC

**DOI:** 10.1093/medlaw/fwac023

**Published:** 2022-07-21

**Authors:** Clayton Ó Néill

**Affiliations:** School of Law, Queen’s University Belfast, BT7 1NN, Northern Ireland, UK

**Keywords:** Care homes, COVID-19, Human Rights, Limitations, Restrictions, Visitation

## Abstract

This article discusses the human rights of residents in care homes in England who were affected by restrictions that were imposed during the first months of the COVID-19 pandemic in order to safeguard health and life at a time of public health emergency. It focuses on the potentially adversarial relationship between the need to protect the health of these residents and the possible adverse interferences with their human rights in the initial phase of the pandemic. The scope and application of these rights to the healthcare context is not straightforward due to the exigencies of the pandemic. Consideration is given to whether their rights, as protected by the European Convention on Human Rights (ECHR) and the United Nations Convention on the Rights of Persons with Disabilities (CRPD) are vindicated or breached by the actions taken in the context of the COVID-19 pandemic. The article questions whether the restrictions that were applied were justified, given the limitations that exist within some ECHR Articles. It deliberates upon what can be done to ensure that relevant bodies and care homes, themselves, are better enabled to respond to a public health emergency in an individualistic, rights-based manner, based upon both principlism and pragmatism.

## I. INTRODUCTION

Year 2020 was a year that was dominated by the COVID-19 pandemic. Words such as ‘quarantine’, ‘social distancing’, and ‘self-isolation’ were part of the everyday parlance. These words, in themselves, sound very lonely and isolating. Indeed, one of the main measures used in preventing the spread of COVID-19 was to try to ensure that people were physically separated from others. This was particularly challenging for those who lived on their own and for those who were vulnerable.

This article focuses on the relationship (or dichotomy) between the need that existed at the onset of the pandemic to protect and safeguard residents of care homes and potential adverse interferences with their human rights. Specific attention is given to the treatment of capacious elderly care home residents in England. Many of these older residents had a range of disabilities. Notwithstanding the fact that people who are not capacious constitute a significant population of those who are residents in care homes, the focus of this article, as illustrated by the vignette of the fictional Janet, reflects only the context of persons with capacity.

Some fundamental questions are addressed here. Were the rights that are protected by the European Convention on Human Rights (ECHR) of residents of care homes violated by the actions taken in the early stages of the pandemic? Or, were identified interferences justifiable, given the extraordinary nature of the times in which we were living and the limitations evident in certain Articles of the Convention? What are the lessons that can be gathered from the way residents of care homes have been treated during this pandemic and what can be done to ensure that the relevant bodies and care homes, themselves, are better enabled to respond to an epidemic in a rights-based manner?

There are four sections in this article. This Introduction comprises Section I. Section II sets out the response to COVID-19 in respect of care homes and provides a summary and analysis of the recent critiques in the Amnesty International report^1^ and the Joint Committee on Human Rights’ (JCHR) report, as well as the responses from the UK government.^2^ Section III assesses the degree to which actions taken were appropriate and whether or to what degree they were aligned with human rights law. Section IV proposes solutions to balancing related conflicting rights. Specific attention is given to the applicability and justiciability of limitations to ECHR Articles in this regard, as well as some provisions in the UN Convention on the Rights of Persons with Disabilities (CRPD).

This discussion regarding potential violations of rights by public authorities recognises that the Human Rights Act 1998 (HRA) gives domestic (and, therefore, direct) effect to certain ECHR rights, including those discussed here. A challenge based on a possible violation of such rights (either because of section 6 of the HRA or sections 3 or 4) is dealt with in a different manner to an assertion that state action has breached international treaties, such as the CRPD. This article does not differentiate between the ECHR as given effect by the HRA and the CRPD in relation to how they apply in domestic law. Neither does it explicitly examine the related role of the Equality Act 2010, which is a domestic statute that could be directly relied upon to allege discriminatory treatment of individuals based on their age or disability. It also provides a meaningful steer as to how challenges to Government policy could proceed before domestic courts. Instead, the article looks more generally at a range of related rights and the translation or otherwise to care home contexts.

Each part of this article is preceded by a short narrative that recounts a small subsection of a small subsection of life, lived and lost in a care home. These narratives are included as a reminder that this article has, at its heart, a human being and it is the contention of this article that, in the fight to protect the health of this (fictional) human being, some humanity was lost. The focus of the article is on older, capacious people in care homes who may have a range of mobility issues. It does not explicitly extend to many others who reside in such contexts, but there may be some parallels that are not fleshed out here.

## II. CARE HOME RESIDENTS AND THE RESPONSE TO THE COVID-19 PANDEMIC IN ITS EARLY STAGES

*Janet lives in the Rainbow Care Home in Nottinghamshire. She is 88 years old. A number of years ago she had a fall and broke her hip, leaving her with significant mobility issues. Two years ago, in conjunction with her niece, who is her next of kin, she made the difficult decision to go to live in the Rainbow Care Home. For the most part, she has been enjoying her life there—enjoying the chats with people of the same vintage and the same interests, enjoying the rather delicious food and appreciating the fact that she does not have to prepare it. She benefits from sporadic nursing care and occasional visits from and to the physiotherapist for treatment for her hip. More than anything, she enjoys Tuesdays when her niece comes to visit and take her out to the shops. They have lunch together in a little café in the busy main street. Her niece also visits every other Sunday and, then, they usually visit a coffee shop and go to the local bookshop to look for the latest thriller because both of them enjoy this genre of writing. Life is not exciting for Janet, but it has purpose and meaning. The loneliness attached to being in a care home is peppered with visits from her loving niece and, as well as that, she engages enthusiastically (and sometimes not that enthusiastically) with the activities organised by the care home. So, her life is brightened by fine painting, nail polishing, knitting, attendance at prayer services, art classes and the formal one-to-one conversation sessions she has once a week with one of the carers.*

*And, so, this not uninteresting life continued until March of 2020…….*

### A. Amnesty International and the Failure to Protect Care Home Residents

Care homes have been at the centre of debate and action and, arguably, inaction in the fight against Coronavirus. The provisions for upholding a satisfactory standard of care in care homes in England fall under the remit of several departments and organisations. The Secretary of State for the Department of Health and Social Care (DHSC) oversees the National Health Service (NHS) and has a duty to prevent inequity.^3^ The bodies in charge of the delivery of services are NHS England and NHS Improvement and Clinical Commissioning Groups (CCGs). Local authorities must act in accordance with the Care Act 2014. There are two types of care homes: residential homes and nursing homes. Some care homes are both residential and nursing homes.^4^ The Care Quality Commission (CQC) and the Local Government and Social Care Ombudsman oversee and audit the care homes.

Many of these care homes are under private ownership but, as Amnesty International rightly points out, this ‘does not in any way lessen the UK government’s obligation[s]’.^5^ These includes the duty to protect against human rights abuse by third parties, including business enterprises’. According to the Office of the United Nations High Commissioner for Human Rights (OHCHR):

States must protect against human rights abuse within their territory and/or jurisdiction by third parties, including business enterprises. This requires taking appropriate steps to prevent, investigate, punish and redress such abuse through effective policies, legislation, regulations and adjudication.^6^

In some cases, care homes have acted with great urgency, propriety and attention to patient safety and protection during this pandemic. Sometimes, this has occurred with insufficient personnel, resources, and Personal Protective Equipment (PPE). At other times, evidence suggests that a number of care homes have not been sufficiently diligent in preventing and halting the spread of Coronavirus.^7^ In both contexts, people have died. In both contexts, some would suggest that the response of the government has, particularly in the initial stages, been of limited benefit and limiting success.^8^

Let me briefly set out the initial approach in England to attempting to stop the spread of COVID-19 in care homes. The approaches adopted in the devolved nations are not addressed due to different timescales and variety of strategies adopted. Essentially, residents in England were not allowed to leave their care home to visit family or to engage in external recreation activities. A range of restrictions was put in place in terms of limiting visitors to care homes, in accordance with lockdowns and tiers and rate of spread of the virus. Care homes were given a number of supports to allow them to implement COVID-19 strategies. For example, a support package for care homes accompanied by a £600 million Adult Social Care Infection Control Fund was introduced by the government on 15 May 2020.

Diverging approaches were adopted in respect of the ordinary person on the street and the care home resident under lockdown. In the community, COVID-19 Regulations were backed by criminal sanctions to promote compliance, but even with the gravest of restrictions, at all stages, a person could, at the very least, take short walks outside their home. The same (restricted) ‘liberty’ did not apply to residents of care homes. Their lives were diminished by the failure to hug, speak to and engage with the people they most loved. Care homes could be lauded for the stringency of their action in preventing the spread of COVID-19 but, arguably, they could also be chastised for limiting the expression of individual liberty and autonomy. Generically applied restrictions were, thus, questionable.

Amnesty International’s report, entitled *As if expendable: The UK Government’s failure to protect older people in care homes during the Covid-19 pandemic* describes and vivifies the tragic consequences of COVID-19 in care homes.^9^ Let this sink in: between 2 March and 12 June 2020, 18,562 nursing home residents died in England from COVID-19 and there were 28,186 ‘excess deaths’ in comparison with the same period in preceding years ie a 46% increase, which arguably could have involved undiagnosed COVID-19-related deaths.^10^ In addition, almost 40% of the people who died due to COVID-19 died in care homes.^11^ On 16 March 2020, routine visits by the CQC were suspended and, following this decision, local government and the Social Care Ombudsman stopped all casework activities. Visitors were also banned at the same time, a new reality that was devastating for residents and family members alike. Family members raised concerns about the lack of monitoring. For example, the CQC noted that there was a significant increase in calls pertaining to abuse and staff not wearing PPE.^12^ Amnesty International refers to the ill-effects of prolonged isolation. Even when many restrictions were lifted in July, the government guidelines stated that ‘where visits do go ahead, this should be limited to a single constant visitor, per resident, wherever possible’.^13^ Updated advice in the *Adult Social Care Winter Plan* referred to the need for care home staff to supervise visitors.^14^ It is troubling that this report alleges that the human rights of residents of care homes have been insufficiently considered and protected by governmental action.

Amnesty International refer to the failures on the part of the UK government, as well as national and local-level bodies, to take appropriate action or to adopt robust strategies/policies. Ultimately, Amnesty International argue that the human rights of older people in care homes have been violated—this includes a breach of the right to life, the right to health, the right to non-discrimination, respect for private and family life and, possibly, freedom from inhuman or degrading treatment. The report refers to different examples where care home residents had greater exposure to a risk of contracting COVID-19. The report contends that COVID-19 represented a disproportionate risk to older people and that there was a direct correlation between this level of risk and the failure to take action. The report describes the actions of the UK government as ‘a chronology of failure’.^15^

The systemic failures identified included the following examples: mass discharging of patients, governmental advice that there was no need to wear PPE for asymptomatic cases of the virus and a failure to assess the ability of care homes to cope with and discharge residents who returned from hospital.^16^ The report also discusses the fact that blanket ‘Do not Attempt Cardio Pulmonary Resuscitation’ (DNACPR) orders were issued in some care homes.^17^ In response to this, the British Medical Association (BMA) Royal College of General Practitioners (RCGP) and the CQC and the Care Provider Alliance (CPA) stated that ‘[i]t is unacceptable for advance care plans, with or without DNAR form completion to be applied to groups of people of any description. These decisions must continue to be made on an individual basis according to need’.^18^

In addition, in many cases, care homes failed to receive regular oversight from bodies such as the CQC. Other failures pinpointed by Amnesty International include unequal access to services under the NHS and the fact that many General Practitioners (GPs) were not visiting nursing homes and providing medical assessment, support, and treatment. A significant issue raised by Amnesty International is the fact that the suspension of family and overnight visits had an alarmingly adverse impact on the health and wellbeing of many residents, where the prohibition on freedom resulted, in some cases, in reduced movement, loss of appetite, depression and a loss of willingness to continue living.^19^ The Amnesty International report is difficult to read—its findings relate to real and vulnerable people who, in many instances, have been robbed of their ability to live their lives in a manner that respects their dignity and autonomy.

The JCHR have also published a report on the response to the COVID-19 crisis by the UK government.^20^ This report recognises that the government’s aim in responding to the COVID-19 crisis had been to save lives, in line with Article 2 ECHR.^21^ As the Joint Committee puts it, as a consequence, ‘[m]any have experienced the widest and deepest set of government interferences with their rights in their lifetimes’.^22^ In a similar manner to Amnesty International, the JCHR argue that the blanket application of DNACPRs represented an interference with individual human rights and this should have been explicitly prohibited by the government.^23^

The JCHR address the fact that a public inquiry will be needed to consider how the government handled the crisis and why there have been so many deaths:

… an inquiry should be timely, have focused objectives and be time-limited. This inquiry must consider, at least, deaths in detention settings; deaths of healthcare and care workers and the availability of PPE; deaths in care homes due to early releases from hospitals; and deaths of transport workers, police and security guards due to inadequate PPE.^24^

The JCHR makes the following points in respect of the ‘very high death toll in care homes’.^25^ Factors such as hospital discharge policies to care homes, testing procedures, availability or non-availability of PPE, lack of or insufficiency of data may have contributed to the death rate in care homes.^26^ They contend that the state should interrogate these deaths as a matter of priority, due to the fact that the state has procedural obligations under Article 2 ECHR.^27^ They address the issue of restrictions on visitation and consider that the initial ‘blanket visiting bans for those deprived of their liberty are contrary to the rights of residents and their families under the ECHR’.^28^ Thus, the report asserts that there was a deprivation of liberty implicit in these significant restrictions on visitation, but it does not, however, provide conclusive elaboration on this claim. In terms of reform, the report calls for measures to be introduced that put human rights of care home residents at the heart of decision-making.^29^ The JCHR welcomes updated DHSC guidelines, which support the development of individual policies for care homes under the framework for local areas policies.^30^ They hope that this guidance will allow for a proportionate response to the issue of visiting which ‘minimises any necessary interference with residents’ right to respect for private and family life under Article 8 ECHR.^31^ Therefore, they argue against blanket restrictions on visitation and contend that ‘[r]estrictions on visiting rights must only be implemented on the basis of an individualised risk assessment and such risk assessment must take into account the risks to the person’s emotional wellbeing and mental health of not having visits’.^32^

The UK Government responded to the findings of the JCHR report on 15 July 2021. They asserted that the UK’s response to its positive obligations under Article 2 were appropriate and were aligned to the main goal to protect lives. It noted that visiting arrangements, albeit limited, had been available throughout the pandemic. This government response also determined that visiting restrictions did not contravene Article 8 rights and stated that ‘[w]e have made judgments that balance these rights to enable residents to have meaningful visits with their families and loved ones while ensuring that residents are protected, as far as possible, from infection and harm from COVID-19’.^33^ The UK Government response also pointed to the further guidance that had been issued in mid-2020 which included individual risk assessments, which would allow for proportionate decision-making in relation to visits to individual residents.^34^ However, this individual approach was tightened again during the second wave of the pandemic in the last half of 2020 and in the beginning months of 2021. Additional information as to the appropriateness of restrictions will be made available into the future as part of the government’s response to the human rights implications of a long lockdown and their inquiry into protecting human rights in care settings.^35^

The next part of this article will attempt to explore further potential violations of human rights.^36^

## III. CARE HOME RESIDENTS DURING COVID-19: A VIOLATION OF HUMAN RIGHTS?

*Janet has an iPad in her hand, she can see the face of her niece on the screen, and she can vaguely hear speech in the background, but she cannot engage in a conversation with the screen. The nurses are happy that she can communicate with a family member through this medium or through phone calls, but Janet is sad. She has not seen her niece for over six weeks, they have not had their long chats, they haven’t shared their memories. Her niece has not confided in her about the problems in her life and sought her advice. Janet has nothing to buy. She can buy nothing because she cannot leave this care home, this house, this day room, this bedroom, this prison. Janet knows that the woman in the room opposite is in self-isolation. She has got this COVID-19 virus. There is a sense of panic surrounding her. PPE and gloves and masks adorn the almost faceless bodies that now hand her medication, advise her to socially distance, encourage her to wear a mask and forbid her from opening that door and crossing the threshold into a world where freedom abides. The walls are closing in on her and tears come to her eyes as she knows that today there will be no jolly call out ‘Hello, Auntie Janet, put on your coat and let’s head out’. There will be no activities in the day room, there will be no chats with her friends in their adjoining rooms because they are fearful, tremulous, and nervous. The day is long, the day is dreary, the day is endless. Janet’s frail body takes a little bit of exercise up and down her room. The metal frame thumps on the cold wooden floor. She looks out the window. There is little to see: few cars gravitate past and only the uniforms of carers and nursing staff brighten the doorway as they hurry out and slowly walk in.*

### A. The CRPD: Fixing the Problem or Seeing the Person? 

Many of the residents of care homes come under the remit of the CRPD, which is the ‘first general United Nations human rights convention to expressly protect persons with disabilities’.^37^ When confronted with people with disability, there are two models traditionally used ie the ‘medical model’ and the ‘social model’. According to Paul Harpur, ‘the medical model focuses upon the person with the disability as the problem and looks for cures’ and ‘[t]he social model is far more popular with disability scholars and those interested in the human rights of persons with disabilities’.^38^ In advocating this social model, authors such as Abberley^39^ and Barnes, Mercer and Shakespeare^40^ argue that impairment and disability should be considered as separate entities. They believe that disability emanates from the way in which society is structured to disempower those who have impairments.^41^ The treatment of people with disabilities in care homes during COVID-19 appears to reflect the medical mode of looking at disability, rather than the more inclusive social mode.

The CRPD provides guidance on what interventions are required to ensure that persons with disabilities can exercise their human rights. Article 1 summarises the purpose of the CRPD as being ‘to promote, protect and ensure the full and equal enjoyment of all human rights and fundamental freedoms by all persons with disabilities, and to promote respect for their inherent dignity’. Articles 3–9 of the Convention, which include ‘universal rights’, and Articles 10–30, which include ‘substantive rights’ provide protection to people with disabilities.^42^ Such rights reinforce rights that already exist and some rights in the CRPD are constructed to provide for the realisation of well-established rights that are protected elsewhere. For example, Harpur points out that well-established rights include the right to life and prevention from cruel, inhuman or degrading treatment.^43^ These civil and political rights are given protection in, for example, the Universal Declaration of Human Rights (UDHR) and, in the European context, the ECHR. To ensure that these well-established rights are vivified for people with disabilities, the CRPD includes rights that have singular relevance to this group of people. These include the following rights: ‘the rights to respect for home and the family, to healthcare, to habitation and rehabilitation and to work, and to an adequate standard of living and social protection’.^44^

Harpur argues that concentrating on ‘fixing’ people with disabilities has caused ‘inferior and exclusionary policies’.^45^ The restriction of visitation to residents of care homes, aimed at fixing the problem of the spread of COVID-19 in this vulnerable population, and the new rules imposed upon these residents may, in time, be found to be discriminatory. At the very least, the restrictions imposed reflect an application of the medical view of disability, which sees disability as a problem to be fixed, rather than the more empowering social model. The issue here is not so much whether measures for people living in care homes protected their lives judiciously but whether these protections were so invasive and so restrictive as to prohibit ‘human thriving’^46^ and the possibility of living ‘the Good Life’: ‘[t]he idea of the Good Life—of what constitutes human thriving—is, implicitly, the foundation and justification of the law’.^47^ It is arguable that the restrictions that were imposed may have been overly focused on safeguarding the biological personhood and may have negated the fact that ‘[o]ur moral value (our personhood) and our identities are a complex mix of biology, psychology and relationship’.^48^ The actions taken to support the commendable aim to save lives should have been commensurate with the need of residents in care homes to live those lives with joy and with purpose, enriched by human loving contact. Human rights instruments are constructed to protect the way in which life is lived.

There is potential now for ‘disability rights advocates’^49^ to make use of the CRPD, which reflects the social model of disability, as a tool for change in view of the fact that its provisions will help to demonstrate whether residents of care homes were/are treated differently to other members of the population. It is interesting that the December 2020 DHSC guidelines, which allowed for outward visits, included the need for individual risk assessment and call for explicit supports to be put in place to support the transfer of policy into practice. This is reflective of the spirit of the CRPD. In many cases, residents of care homes are subject to standard authorisations under the Mental Capacity Act 2005 which, in turn, carries certain obligations under Deprivation of Liberty Safeguards (DoLS), which will become Liberty Protection Safeguards. This has the potential to have an impact on the Article 5 (deprivation of liberty) ECHR rights of patients who lack capacity, but DoLS does not apply to the capacious patients considered in this article.

### B. The ECHR: Are the ‘limits of limits’ Reached or Has a ‘fair balance’ Been Reached?

The following section attempts to show whether the response to the COVID-19 crisis in care homes complies with a number of ECHR Articles. Undertaking this task necessitates setting out initially the fact that ‘these challenging times’ have imposed an equally challenging reality. In the well-intentioned rush to protect, to save life, to halt the spread of an invidious disease, to support the vulnerable, there has been a consequent possibility that some human rights have been threatened or denied or questioned or breached or, alternatively, vindicated and, indeed, celebrated. At a time of pandemic, governments and agencies often have to act on the hoof, swiftly, alert to the dangers from without, but perhaps, insufficiently mindful of the dangers from within. This section seeks to look at some of the Articles of the ECHR and their related limitations and to gauge whether violations existed.

It is obvious that most member states show willingness and, indeed, enthusiasm when engaging with Articles of the Convention. Breaches are less easy to prove or quantify, due to the prevailing limitations that apply to some of the Articles. There has been some debate in the European Court of Human Rights (ECtHR) about the curtailment of rights and the legitimacy of restrictions. For example, in *Folgerø and Others v Norway*, the ECtHR has discussed the right to access education, as protected by Article 2 of Protocol Number 1 of the Convention. It has determined that there are instances when restrictions may be imposed, but such restrictions should not be so constructed as to curtail the right to such an extent that this would result in impairing the essence of the right.^50^ And that is the crux of the matter here: when limits are placed on rights, as has happened to residents of care homes for what are arguably good societal and medical reasons, are these limitations so restrictive as to impair the essence of the right protected? Were the measures imposed the least restrictive measures possible in accordance with the spirit of the Convention?

Janneke Gerards shows that for a restriction of an ECHR right to be warranted, ‘it is not enough for it to have a sound legal basis and to pursue a legitimate aim. Restrictions of Convention rights also must be shown to be necessary or proportionate, and there must be a fair balance between the aim being served and the right being restricted’.^51^ The ECtHR has signified in *Soering v the United Kingdom* that ‘inherent in the whole of the Convention is a search for a fair balance between the demands of the general interest of the community and the requirements of the protection of the individual’s fundamental rights’.^52^

Thus, a potential conflict of rights exists: the right to life of care home residents and the right to life of those within the broader population. It is arguable that decisions regarding the discharge of COVID-19 patients back to care homes potentially violated their right to life. This action, of course, comes down to individual hospital practices and it is not clear that specific action on the part of the state contributed to the practice of discharge. As the JCHR report notes, there was guidance from the government which could be linked with the decisions of hospitals regarding discharge of care home residents with COVID-19 which may, in turn, engage its operational obligation to protect. An examination as to how challenges to Government policy could proceed before domestic courts could also, arguably, consider the Equality Act 2010 which is a domestic statute that could be directly relied upon to allege discriminatory treatment of individuals on the basis of their age or disability.

Significant numbers of hospital patients who had contracted COVID-19 were sent to COVID-19 free care homes where the rate of COVID-19 transmission subsequently increased apace.^53^ The statistics bear harrowing reading and can be summarised in one bleak statement: too many older and vulnerable people died in care homes.

Is there, therefore, a conflict of rights between the right to life of care home residents and the right to life of those within the broader population? Can it be inferred that those decisions regarding the discharge of COVID-19 patients back to care homes potentially violated their right to life? Discharge protocols come down to individual hospital practices unless it can be shown that there was something on the part of the state that contributed to the practice of discharge. As the JCHR report notes, there was guidance from the government which could be linked with the decisions of hospitals regarding discharge of care home residents with COVID-19 which may, in turn, engage its operational obligation to protect. Thus, the operational obligation to protect has arisen and, relatedly, a violation of Article 2 could arise on this basis. A separate obligation on states under Article 2 exists. There has been a potential violation given that this obligation requires, in the healthcare context, that states put in place regulations compelling hospitals to adopt appropriate measures for the protection of patients, including a framework that does not permit the discharge of COVID-19 patients from hospitals to care homes.^54^

Article 2 is a seminal right in any discourse pertaining to the restrictions applied to care home residents that were imposed in an attempt to protect life during the COVID-19 crisis. The context of a pandemic is not specifically described in Article 2(2)’s limitations. Foster contends that Article 2 ‘imposes on States an obligation to take steps to protect life’.^55^ This is a positive obligation, which is focused on both the individual and the wider community.^56^ It is interesting that the guidance from the ECHR in respect of engagement with Article 2 refers to the fact that states have not only to refrain from unlawful taking of life, but they must also do all they can to safeguard life.^57^ It is recognised, therefore, that a state has positive and procedural obligations under Article 2. To date the focus has been on what the state can ‘reasonably’ be expected to have done in the circumstances and the ECtHR has repeatedly held that an ‘impossible or disproportionate burden must not be imposed on the authorities’.^58^ This is quite important in the context of the COVID-19 pandemic as, arguably, what the state can *reasonably* be expected to have done is a different standard to all that they could have done’.^59^ This requires states to impose regulations in terms of healthcare that will adequately address the attainment of this right.^60^ The challenges this requirement imposes have been played out in ECtHR jurisprudence, including *Aydoğdu v Turkey*.^61^ Positive actions set out by states should include determining what can be undertaken so as to comply with the provisions of Article 2 and the Convention’s spirit.^62^

Some of the debates in ethics and law as they relate to COVID-19 can be conceived of as being a contest between the values inherent in Article 2 and the provisions in Article 8. Article 8 is the most elastic of the Convention articles: broadly read, it gives a right in Article 8(1) (qualified by reference to broader societal considerations in Article 8(2), to live one’s life as one chooses).^63^ It is entirely improbable to think that the restricted life imposed upon residents in care homes accords with the freedom to ‘live one’s life as one chooses’. Foster argues that, rightly or wrongly, policymakers only seem to consider Article 2.^64^ The whole shape of Article 8, for instance, is determined by the tension with which it co-exists with (particularly) Article 2. The vital connection between the jurisprudence of Article 8 and the jurisprudence of Article 2 is shown very clearly in, for example, the assisted suicide and related cases adjudicated both in Strasbourg and in England.^65^

It is worth noting that the limitations of Article 5(1)(e) explicitly include the control of infectious diseases while Article 8(2) simply refers to ‘the protection of health or morals, or for the protection of the rights and freedoms of others’. At one glance, it would appear that there was no breach of Article 8 in the early restrictions imposed upon residents because it seems clear that the limitations here allow for violation from the Article when the health or freedoms of others are threatened or undermined. But, once again, there is a potential violation in so far as an older person or a vulnerable person has a right to respect for family and private life: engaging in family interaction is a right that is identifiable and applicable and measurable. The fact that it is limited by the need to protect the health and freedoms of others does not negate or annul the right. It just means that the right can be limited in certain circumstances. The need to stop the spread of COVID-19 was, quite obviously, one such applicable circumstance. Actions taken to this spread included primarily prevention or limitation of family visits, the encouragement of Skype/Facetime/iPad related phone calls, which many vulnerable people could not use effectively.^66^ Care homes may have been constrained from taking the option of using protective screens or plastic pods, which might have facilitated greater social interaction. Therefore, the answer here is, once again, that there may not be an explicit breach and that the limitations justify the actions taken. However, the potential for a breach exists and those who implement policy in respect of care home residents should continue to be mindful of this potential. Herring alerts us to the fact that Article 8 contains both positive and negative aspects.^67^ In respect of negative terms, he reminds us that the state must not ‘interfere with an individual’s private or family life unless to do so is necessary under the terms of Article 8(2)—for example, it is necessary to protect the interests of others’. Of course, it is recognised that the restrictions imposed during the pandemic have been done so with a desire to protect the health of all, in line with this obligation. However, Herring also brings to mind the positive obligation that applies under Article 8: ‘[t]his requires that the state, on occasion provide service or otherwise act in a way to enable a person to maintain a family relationship’.^68^ This positive obligation, according to Herring, is limited and ‘a state is only required to take reasonable steps’.^69^ Unfortunately, in the case of the COVID-19 restrictions, few such steps, limited or otherwise, were taken and the state made little effort to comply with this positive obligation. This inaction ignores the competing obligation to protect life under Article 2 which arises when the state knew or ought to have known of a real and immediate risk to life and that arguably is the case in the context of care home residents during the pandemic.

Article 8(1) is easily engaged, but hard (because of the countervailing considerations in 8(2)), to breach. A clash between the right to life of the broader public and the right to private life of care home residents can exist here, leading to potential conflict. But there is also a more central issue regarding the potential conflict between a care home resident’s right to life and their right to private life; care home residents were, for myriad reasons, especially vulnerable to COVID-19 and the restrictions placed on their private life could be said to have been a proportionate interference in order to secure their right to life (ie these steps were necessary to protect the individual residents’ lives). Although I have earlier contended that the state has ‘made little effort to comply’ with its positive obligation to take reasonable steps to protect private life, I recognise that this ignores the competing obligation to protect life under Article 2. This obligation arises when the state knew or ought to have known of a real and immediate risk to life and that, arguably, is the case in the context of care home residents in the midst of the pandemic. There is a consequent potential issue of deprivation of liberty under Article 5 of the Convention in this context.

Many of the residents of care homes may already, however, have been subject to restrictions as part of a standard authorisation and DoLS. The Mental Capacity Act 2005 permits deprivations of liberty subject to the DoLS (which will become Liberty Protection Safeguards in April 2022). However, there are many residents in care homes who were not subject to standard authorisations, and, from the onset of the pandemic, these residents were prohibited from leaving the institution in which they were living. The purpose of this deprivation of liberty was twofold: to maintain their health and to maintain the health of the broader population by preventing the spread of the disease from the vulnerable population to the community. Article 5 protects liberty, but there are limitations attached to this Article. Regarding COVID-19, Article 5(1)(e)’s limitations are relevant. The provision states that liberty can be limited in the context of ‘the lawful detention of persons for the prevention of the spreading of infectious diseases, of persons of unsound mind, alcoholics or drug addicts or vagrants’. Although it follows from this that no explicit breach is evident, many residents in care homes, who were primarily old and vulnerable, were not allowed to leave their institution. Some were self-isolating and could not even leave their rooms.

In *P v Cheshire West and Chester Council* and *P and Q v Surrey County Council*, courts were asks to decide whether the care given to three people with disabilities was depriving them of their liberty and, if this were the case, whether sufficient safeguards had been in place.^70^ The court originally found that the supervisory measures that had been implemented were in line with their best interests. A number of years later the case was taken to the Supreme Court where it was found that the fact that a person had a learning disability did not mean that the right to liberty and its related safeguards could be applied differently. Universality is the key concept or, as Lady Hale states, ‘if it would be a deprivation of my liberty to be obliged to live in a particular place subject to constant monitoring and control, only allowed out with close supervision and unable to move away without permission even if such an opportunity became available then it must also be a deprivation of a disabled person’.^71^ Arising from this case, the restriction of liberty is now determined by an ‘acid’ test which is comprised of two key questions: (1) ‘is the person subject to continuous supervision and control?’ and (2) ‘is the person free to leave?’.^72^ This case has implications for older capacious people in care homes during the COVID-19 crisis whose liberty was constrained. Would the constraints meted therein pass or fail the acid test? Constant supervision and control were *de rigeur* and older people could not leave on a temporary basis to meet their family if that were their wish. Some were able to leave their room but could only take short walks in the corridors and may not have been permitted to enter the communal day room. Most were not permitted to visit other residents and, so, were deprived of valuable human-to-human contact. These residents may have been deprived of liberty with the best of motives and the best of intent, but that good intent still does not annul the fact that the exercise of a human right was limited, even if justified by Article 5(1)(e).

Additional Articles, such as Articles 3 and 14, which deal with the prohibition of torture, inhuman and degrading treatment and discrimination are also relevant here. The definition and understanding of torture, as is understood in Article 3, is set at a very high threshold. Many examples in case law illustrate instances where cases have been shown to fail to reach this limit.^73^ There is no case law to indicate that not allowing visitors to visit care homes constitutes torture or inhuman treatment. The threshold for a breach of Article 3 is set at such a high level that it would be difficult for a court to find such a breach except in extreme situations.^74^ No exceptions or limitations apply to Article 3, which has been considered to be an absolute right. In summary, Article 2 contains express limitations under Article 2(2), whereas Article 3 has no such limitations.

There is no visible conflict between Article 2 and 3 in these circumstances. In the first place Article 2 defends the rights of individuals in the community. This requires that the state do that which is reasonably expected of them. This cannot conflict with Article 3 provisions since inflicting inhuman or degrading treatment would not be considered reasonable in those circumstances. In the second place, the imposition of significant restraints on a person’s liberty and autonomy arguably engages the state’s *negative* obligation not to inflict inhuman or degrading treatment under Article 3. As Article 3 is truly absolute—that is, there are no exceptions to it—then the infliction of inhuman or degrading treatment can never be justified, no matter how strong the competing interest. Thus, there is no conflict here.^75^

Article 14 ECHR protects against discrimination on the basis of a number of protected characteristics, such as sex, race or religion. Interestingly, Article 14 does not refer to discrimination on the basis of age or, indeed, vulnerability. But these two factors may be covered under ‘birth or other status’. In *Glor v Switzerland*, private life within the meaning of Article 8 included the physical integrity of a person.^76^ Age and disability are covered by Article 14.^77^

For a violation of Article 14 to be proven, this violation must, however, be aligned with the breach of another Article. The restrictions imposed on residents of care comes can possibly come under the requirement of ‘necessity’. In this regard, discrimination contrary to Article 14 may be justified if such discrimination is deemed to be necessary. As *Handyside v the United Kingdom* makes clear, there are different standards of necessity for different Articles in the ECHR and ‘absolutely necessary’ is not the standard generally applied, for instance, to Article 8. Discrimination contrary to Article 14 may be justified if it is necessary in the context of Article 14 specifically.

The application of a limitation to Convention rights must be deemed to be ‘necessary to achieve a legitimate aim’. The ECtHR does not permit this determination of ‘necessity’ with ease or with lack of gravitas. The court discussed in *Handyside* what constitutes necessity, ranging from ‘indispensable’, ‘absolutely necessary’ and ‘strictly necessary’ as well as ‘to the extent strictly required by the exigencies of the situation’.^78^ Actions should not fall within the scope of ‘admissible’ or ‘ordinary’ or ‘useful’.^79^ This debate concerning necessity is not, however, pitched at the semantic level. Rather, it proposes that action that is taken based on necessity needs to be gravely and seriously considered. The restrictive actions that were taken at the onset of the pandemic probably did emanate from the requirement of necessity at a societal level and came within the umbrella of action that was ‘indispensable’ and ‘absolutely necessary’. This is a probable rather than a finite assertion and the limitations cannot be set in finite stone, but should move as necessity moves and be amended as necessity moves in accordance with changes in COVID-19 circumstances.

This societal necessity needs to be balanced with the individual needs of human beings who long to thrive,^80^ to reach their own self-fulfilment.^81^ Gerards argues that:

A ‘fair balance’ does not always imply an actual choice to be made between conflicting rights and interests, in the sense that one interest or right has to prevail over another one. Instead, it may be important to look for reconciliation or for a middle ground.^82^

Örücü talks, however, about some actions as reaching ‘the limits of limits’.^83^ For some lonely residents in care homes who have not seen or physically held their relatives for months on end, this point of extremity of limits may well have been reached. No balancing of societal rights and individual rights can or should, in their view, negate the need of residents in care homes for emotional nourishment that is principally wrought by human contact with those they love.^84^

The issue of whether the response to the pandemic in terms of limiting visits to people in care homes was proportionate is, for the moment, impossible to quantify. If a case is taken querying a potential beach of Convention rights, the courts will have to decide whether the interference with rights was proportionate to the legitimate aim pursued.^85^ In this case, the protection given to health and life will be considered to be a legitimate aim and proportionality will be judged based upon the background circumstances, the rights in question and the type of interference that applies.^86^ The court has used different terms which suggest that the rights in the ECHR should take priority and that individual states need to justify any potential interference. This means that, if a case were taken, the Convention rights will be given priority and it will be up to the state to provide a justification for what might appear to be infringements of rights through actions such as significant restrictions on visitation. However, making these determinations may become quite complex because the ECtHR’s decisions have shown that they can align proportionality with the need to balance rights and exemptions. So, thus, a conflict can exist between priority to rights and balancing of rights, particularly as this applies to the scope of the margin of appreciation.^87^ The philosophical argument applies here: a rights-based approach may prioritise rights and a more utilitarian approach and a medical viewpoint of disability might lean towards balancing rights.

The primacy that is given to Article 2 is not contested here. However, it is worrying that it appears that the provisions and limitations that apply to other related Articles have been inadequately considered by those who make decisions for these residents. It is accepted that we have been living in extraordinary times, but that is no excuse for the implementation of policies and practices that seem to have the potential to interfere adversely with human rights.

## IV. A NEED FOR A RIGHTS-BASED INDIVIDUALISTIC APPROACH

*Janet didn’t get COVID-19, she did not succumb to the disease that had captured the life of her friend, Mary. So, she was not a victim of this epidemic and will not be included in the daily mortality figures. She will be sad about that because her death, (yes, she died), happened because COVID-19 happened. She lost her grip on life and on the purpose of life. Just as COVID-19 took a grip of the consciousness of those outside the portals of the care home and those within its structure, she found the loneliness hard. She found the hunger for purpose difficult. She found a life without love unforgiving in its brutality. She missed her niece, she missed the life she had before all this COVID-19 happened and, so, almost unknown to herself, she began to slip away: one meal less, one drink less, until, finally, there were no meals at all, no drinks at all and no enforced nutrition, thankfully, since years ago she had forcefully decided against this. So, her death bed came, and the nurses were good and caring and the end-of-life team could not be faulted. She felt the iPad in her hand again and someone saying to her ‘Here’s your niece, speak to her’ and Janet was able to mumble a few sentences to say some kind of goodbye and she heard her niece’s voice in the background. The love travelled over the distance, not tangible as the grasp of a hand would have been, not visible as a loving caress would have been, not real as the presence in the room would have been, but tangible, nonetheless. And she slowly slipped away in that lonely busy room where the cold professionalism of care wore PPE and held a stethoscope and silently mouthed the words ‘She is gone’. And the undertaker came, and she was buried—no Mass, no wake, no kindly visits from neighbours bearing casseroles. Just another number which will not be counted in the figures that count.*

What is required to limit potential breaches of the rights of residents of care homes in a time of public health emergency is a principled, pragmatic and individualised response that also considers harm caused. This approach would help to ensure that a fair balance exists between protecting residents from the harm of COVID-19 and also ensuring that their autonomous rights are protected in communal living contexts.

### A. Principled and Pragmatic Response

Applying core principles of ethics in a pragmatic manner is the first step in finding a fair balance between competing rights. Beauchamp and Childress adopt a principled-based approach, which constitutes a dominant approach to the application of medical ethics. The four principles are autonomy, beneficence, non-maleficence and justice.^88^ They are ‘the most general and basic norms’ but the application of these general norms need to be refined based on the specific scenarios.^89^

*Autonomy* is about making one’s own choices in the context of ‘circumstantial freedom’.^90^ Autonomy is not limitless; it is facilitated by the environment and by others. Any restrictions imposed on individual autonomy need justification. In other words, the choices of others cannot be interfered with unless doing so is justified. Healthcare providers must treat people like they are capable of choice and must not be ready to assume incompetence. There is, certainly, evidence that the restrictions imposed upon capacious residents in care homes represented a breach of their autonomy and a dulling of their autonomous voice. Insufficient attention was, therefore, given to the key principle of autonomy.

*Non-maleficence* is about not inflicting harm on others either through negligence or deliberately. It is innately vivified in the Hippocratic Oath—first do no harm! There is no doubt that some of the approaches adopted caused harm to residents of care homes. The most noteworthy harm was manifested in the fact that a significant number of residents died from COVID-19 during the early stages of the pandemic, partly because of the discharging of patients from hospitals to care homes without COVID-19 testing. The lack of appropriate PPE likely also increased the rate of COVID-19 transmission in these settings and, thus, caused harm. The later policies adopted in relation to social isolation undoubtably caused injurious harm to people who were bereft of physical and social contact with family and other residents/friends in the care homes. Beauchamp and Childress say that non-maleficence is balanced with the related principle of *beneficence*. Beneficence concerns the achievement of what is best for each individual. Many of the policies adopted were beneficent in their aim but, perhaps, not in application and in the consequences that ensued. This involves consideration of the question: benefit for whom?

*Justice* is about transparency and fairness. Emily Jackson talks about the interpretation of justice as treating people in a like-for-like manner.^91^ If the treatment of residents in care homes in the initial stages of COVID-19 is compared to that of the community population, then it is apparent that like-for-like treatment did not apply and the application of justice was, consequently, flawed.

Ethical principles are at the heart of medical ethics and the approaches adopted at the start of the pandemic were insufficiently grounded on these principles. In general, action preceded serious ethical consideration. A measure of ethical preparedness for future endemics/pandemics is, consequently, necessary. That preparedness should include ensuring that these four principles, at the very least, provide steer for policy making and action, particularly in the context of public health frameworks.

Practical realities, however, apply with vicious force in times of healthcare emergencies. Principlism alone, therefore, will not resolve the conflict between the need to protect both life and autonomy. Resolving this ethical dilemma must also include the use of pragmatism: the pragmatic eye on the moral dilemma. Pragmatism seeks to problem-solve and, in the early stages of the pandemic, there was limited problem-solving on an individual basis. The application of pragmatism to challenging contexts would give another tool to decision makers, namely a practical wisdom where solutions are found to complex problems based upon individual circumstances. Those who make decisions about restrictions would apply pragmatism as being ‘the right way of acting in difficult and uncertain circumstances for a specific end’.^92^ This does not mean abandoning principlism or a universal moral foundation. Pragmatic methods consider and act upon principled justification for actions taken. Resolving ethical dilemmas associated with the pandemic into the future will involve the application of practical wisdom so that just decisions are made for and with residents in a courageous and principled manner.

### B. Individualised Response and Consideration of Harm

Plato conceives of an ideal society that is governed by attributes such as wisdom, temperance, and justice.^93^ The individual and society exist in a form of organic unity, where the conditions of one are directly related to the other. Both form parts of a symbiotic whole where the actions that benefit society must also benefit the individual. The corollary is also true—the actions that benefit the individual need also to benefit the community at large. Herein lies one solution to the balancing of conflicting rights: adopting a Plato-esque response, I suggest that the actions taken to benefit society at large, such as prohibiting visitors in care homes, must also benefit the individual.

One case that illustrates the need for an individualised approach was *BP v Surrey County Council*.^94^ This case related to a conflict between the wishes of one member of the family of a care home resident who was living with Alzheimer’s disease (and had a range of disabilities that restricted his ability to communicate) and the wishes of the care home. The family member wanted to take her father (BP) home to reside with her because the restrictions were having a gravely negative impact upon him. The care home and its medical representatives determined that it would not be appropriate for BP to leave the care home. The case came before the courts on two occasions. It was first determined that, as long as good attempts were made to use technological means of communication, then the most appropriate means of supporting BP was for him to stay in the care home. The second judgment, however, proposed that BP could return to live with his daughter due to some changing circumstances.^95^ The significance of this case lies in the fact that the court determined that COVID-19 restrictions were causing harm to BP: he no longer received visits from family and friends and the visits of his Mental Capacity Assessor stopped. In the first judgment, Hayden J was concerned about possible interference with Article 11 CRPD which holds that state parties shall take ‘all necessary measures to ensure the protection and safety of persons with disabilities in situations of risk’. Hayden J also addressed Article 5 ECHR implications and concluded that the state must take reasonable steps to prevent a deprivation of liberty. Hayden J’s second judgement, again, recognised harm: the separation of BP from his home caused depression. This judgment balanced the results of professional assessment and best interests rather than placing a central focus on professional assessment alone. It clarifies that there had been changes in the health of BP in the intervening period and that the parties were in agreement that BP was now free to leave the care home. It can, thus, be argued that the decision to permit him to change abode was less a matter of the court making a determined effort in establishing best interests and more a consideration that the medical professionals and his family knew what was in BP’s best interests.

Resolving the conflict between restricting the spread of COVID-19 and retaining liberty of residents involves seeing each individual within the communal context and advocating for them in light of their particular needs and rights. For example, it may be appropriate for Joe to leave the confines of the care home, given particular circumstances, but the same decision might not be appropriate for Jill, in light of her individual circumstances. The approaches adopted in the initial stages of the pandemic caused the individual voices of residents to be diluted. The fact that the pandemic was used as a justification to interfere with human rights cannot negate the fact that the principle of autonomy was side-lined. An individualised approach would be rights-based and would seek to give parity of esteem to the individual within the communal.

Aligned to this individualised approach, it is also apparent that policy makers and healthcare administrators needed also to consider the potential harm associated with the imposition of restrictions. In fact, Usher considers that several principles in addition to those articulated by Beauchamp and Childress, including harm, should be applied in public health contexts.^96^ Accordingly, the risk of death should be weighed against the risk of harm caused by social isolation and other harmful consequences of the COVID-19 measures adopted. Strict communally applied restrictions on visitation were not appropriate because they did not respect or identify a need to assess degrees of harm. People are not homogonous: the harm that can befall them because of COVID-19 needed to be weighted on an individual basis so that other factors, and the harm that they could cause, were also considered.

Ultimately, the deprivations associated with restrictions on liberty in the initial stages of the pandemic were excessive. In my view, there is little evidence that any serious attempt was made to engage with related rights reconciliation. In dealing with future iterations of this pandemic or of other public health emergencies, greater attention will need to be placed upon respecting the rights of the individual and acknowledging and attempting to minimise the harm or potential for harm that well-intentioned actions might have on care home residents.

## V. CONCLUSION

It is the court of law in future judgments that will pinpoint whether and to what degree human rights were compromised in these challenging circumstances. Notwithstanding that caveat, this author takes a stance that the rights of residents in care homes were not protected with appropriate care, caution, courage, and concern. While there was a need to protect life and the restrictions on visitation might have justifiable, for the most part, individual autonomy was not upheld sufficiently. That is not something of which we can be proud.

The fictional story of Janet is, unfortunately, not all that fictional at all. It reflects the reality that the limitations imposed upon people in care homes have had sad consequences that causes us to question the value that is sometimes ascribed to their humanity.

## Footnotes

1 Amnesty International, *As if expendable: The UK Government’s failure to protect older people in care homes during the Covid-19 pandemic* (Amnesty International 2020) <https://www.amnesty.org/download/Documents/EUR4531522020ENGLISH.PDF> accessed 14 March 2022.

2 House of Lords, House of Commons Joint Committee on Human Rights, *The Government’s Reponses to COVID-19: Human Rights Implication* (21 September 2020) <https://committees.parliament.uk/publications/2649/documents/26914/default/> accessed 14 March 2022.

3 See the Health and Social Care Act 2012.

4 In addition to this, day care facilities can sometimes act as nursing homes and can have this classification. This article does not, however, consider day care facilities because most of the issue raised here are manifest in different ways in those facilities, if at all.

5 Amnesty International (n 1) 46.

6 OHCHR, *Guiding Principles on Business and Human Rights: Implementing the United Nations ‘Protect, Respect and Remedy’ Framework* (United Nations 2011) <https://www.ohchr.org/Documents/Publications/GuidingPrinciplesBusinessHR_EN.pdf> accessed 15 March 2022.

7 JCHR (n 2) 33.

8 ibid; Amnesty International (n 1).

9 In terms of methodology, Amnesty International interviewed a range of different people, such as relatives, nursing home managers and staff and medical personnel. Residents were not interviewed because, in the Covid-19 climate, they could not be conducted in-person.

10 Office for National Statistics, ‘Deaths involving Covid-19 in the care sector, England and Wales’ data, Table 1 and Table 6 <https://www.ons.gov.uk/peoplepopulationandcommunity/birthsdeathsandmarriages/deaths/articles/deathsinvolvingcovid19inthecaresectorenglandandwales/deathsoccurringupto12june2020andregisteredupto20june2020provisional> accessed 11 March 2022.

11 ibid, Amnesty International (n 1) 5.

12 CQC, ‘Regulating During COVID-19—Why Raising Concerns more Important than Every’ (CQC 17 June 2020) <https://www.cqc.org.uk/news/stories/regulating-during-covid-19-why-raising-concerns-about-care-more-important-ever> accessed 11 March 2022.

13 The most recent guidelines: Department of Health and Social Care, *Guidance: Visiting Care Homes During COVID-19* (updated 1 December 2020) <https://www.gov.uk/government/publications/visiting-care-homes-during-coronavirus/update-on-policies-for-visiting-arrangements-in-care-homes> accessed 11 March 2022.

14 ‘Adult social care: our COVID-19 winter plan 2020 to 2021’, 18 September 2020, <https://www.gov.uk/government/publications/adult-social-care-coronavirus-covid-19-winter-plan-2020-to-2021/adult-social-care-ourcovid-19-winter-plan-2020-to-2021> accessed 11 March 2022.

15 Amnesty International (n 1) 15.

16 ibid 6.

17 ibid 7.

18 Joint statement by the British Medical Association (BMA), the Care Provider Alliance (CPA), the Care Quality Commission (CQC) and the Royal College of General Practice (RCGP), 1 April 2020 <www.rcgp.org.uk/about-us/news/2020/april/joint-statement-on-advance-careplanning.aspx> accessed 11 March 2022.

19 Amnesty International (n 1) 8.

20 JCHR (n 2).

21 ibid.

22 ibid 5.

23 P Harpur, ‘Embracing the New Disability Rights Paradigm: The Importance of the Convention on the Rights of Persons with Disabilities’ (2012) 27(1) Disability & Society 1–14, 4.

24 JCHR (n 2) 6.

25 ibid [96].

26 ibid.

27 ibid [97].

28 ibid [136].

29 ibid.

30 ibid.

31 ibid.

32 ibid.

33 UK Government, ‘Care homes: Visiting restrictions during the covid-19 pandemic: Government Response to the Committee’s Fifteenth Report of Session 2019–21’ <https://publications.parliament.uk/pa/jt5802/jtselect/jtrights/553/55302.htm> accessed 15 March 2022.

34 ibid.

35 UK Parliament, Joint Committee on Human Rights, ‘New Inquiry: Protecting Human Rights in Care Settings’ (21 September 2021) <https://committees.parliament.uk/committee/93/human-rights-joint-committee/news/157644/new-inquiry-protecting-human-rights-in-care-settings/> accessed 15 March 2022.

36 The term ‘derogation’ is used to refer to formal steps taken by the state to depart from its rights obligations in times of war or public emergency. Thus, the terms ‘violation’ and ‘breach’ are used interchangeably here.

37 Harpur (n 23).

38 ibid.

39 P Abberley, ‘The Significance of Work for the Citizenship of Disabled People’ <https://disability-studies.leeds.ac.uk/wp-content/uploads/sites/40/library/Abberley-sigofwork.pdf> accessed 11 March 2022.

40 Colin Barnes, Geof Mercer and Tom Shakespeare, *Exploring**Disability: A**Sociological**Introduction* (Polity Press 1999).

41 Harpur (n 23) 3; M Oliver, *The**Politics of**Disablement* (Macmillan 1990) 11.

42 Harpur (n 23) 4–5.

43 ibid 4–5.

44 ibid 5.

45 ibid 11.

46 C Foster and J Herring, *Human Thriving and the Law* (Springer 2018) 87.

47 ibid vii.

48 C Foster and J Herring, *Identity, Personhood and the Law* (Springer 2017) 70.

49 See Harpur (n 23) 11.

50
*Folgerø and Others v Norway*, ECtHR (GC) 29 June 2007, application no 15472/02, para 98 See J Gerards, *General Principles of the European Convention on Human Rights* (CUP 2019) 253. Also, see *Hasan and Eylem Zengin v Turkey*, ECtHR 9 October 2007, application no 1448/04, para 73; *Catan and Others v Moldova and Russia*, ECtHR (GC) 19 October 2012, application no 43370/04, para 140; *Velyo Velev v Bulgaria*, ECtHR 27 May 2014, 16032/07, para 32; *Çölgeçen and Others v Turkey*, ECtHR 12 December 2017, application no 50124/07, para 49.

51 Gerards, ibid 229.

52
*Soering v the United Kingdom*, ECtHR 7 July 1989, application no 14038/88, para 89; see Gerards (n 50) 229.

53 See JCHR (n 2).

54 See *Lopes de Sousa Fernandes v Portugal* Application no 56080/13, Grand Chamber, 19 December 2017.

55 C Foster, ‘We’re all Vitalists Now’ (*Practice Ethics*, 19 May 2020) <http://blog.practicalethics.ox.ac.uk/2020/05/were-all-vitalists-now/> accessed 11 March 2022.

56 See *LCB v the United Kingdom* (1999) 27 EHRR 212; *Calvelli and Ciglio v Italy* (application no 32967/96; [2002] ECHR 3), para 49 and *Vo v France* (2005) 40 EHRR 12, para 89.

57 Guidance on art 2 of the European Convention on Human Rights. <https://www.echr.coe.int/Documents/Guide_Art_2_ENG.pdf> accessed 11 March 2022.

58 See, the guidance document at, for instance, [19] and [35].

59 ibid 11.

60 ibid 8. The guide states that ‘[i]n broad terms, this positive obligation has two aspects: (a) the duty to provide a regulatory framework; and (b) the obligation to take preventive operational measures’.

61
*Aydoğdu v Turkey* (application no 4044906, 30 August 2016).

62 See Miroslav Baros, ‘The UK Government’s Covid-19 Response and Article 2f the ECHR (Title 1 Dignity; Right to Life; Charter of Fundamental Rights of the EU)’ (2020) 9(3) Laws 19.

63 Foster (n 55).

64 ibid.

65 See, eg *R (Nicklinson) v Ministry of Justice* [2014] UKSC 38 and *Pretty v United Kingdom* (2002) 35 EHRR 1.

66 JCHR (n 2).

67 J Herring, *Older People in Law and* Society (OUP 2009) 123.

68 ibid.

69 ibid.

70 [2014] UKSC 19.

71 ibid at [46].

72 ibid.

73 See *Selmouni v France* (application no 25803/94); *Ireland v United Kingdom* [1978] 2 EHRR 25 at [162].

74 See Equality and Human Rights Commission, ‘Article 3: Freedom from Torture and Inhumane and Degrading Treatment or Punishment’ in Human Rights Review (Equality and Human Rights Commission 2012) 69–134.

75 According to Lord Kerr in the *Northern Ireland Abortion Case* [2019] 1 All ER 173 at [215]: ‘If the treatment to which an individual is subjected can properly be regarded as torture or inhuman or degrading, it does not matter a whit what the person or agency which is responsible for the perpetration of that treatment considers to be the justification for it. Nor does it matter that it is believed to be Page 95 necessary to inflict the treatment to protect the interests of others. Torture and inhuman or degrading treatment are forbidden. That is an end of it’.

76
*Glor v* *Switzerland* (application no 13444/04). In this case, which deliberated upon the payment of tax and found that the Swiss court had not taken account of the applicant’s physical circumstances, including the constraints of partial disability. The ECtHR concluded that there had been a violation of art 15 and art 8 ECHR. The Court found that the applicant had been the victim of discriminatory treatment because the Swiss state were not able to justify the distinctions made between people who were unfit for military service and not liable to a specific tax and those who were unfit for service but were obliged to pay the tax. The Court suggested that the option of alternative forms of service (including less physical effort) should have been given to people with a disability and not just to be the prerogative of those who conscientiously objected.

77 See, ECtHR, *Guide on Article 14 of the European Convention on Human Rights and on Article 1 of Protocol No**12 to the Convention* (31 December 2020) at [143] and [160]: <echr.coe.int/Documents/Guide_Art_14_Art_1_Protocol_12_ENG.pdf> accessed 11 March 2022.

78
*Handyside v the United Kingdom*, ECtHR 7 December 1976, 5493/72, para 48. See Gerards (n 50) 230.

79 Gerards (n 50) 230.

80 Foster and Herring, *Human Thriving and the Law* (n 46).

81 C Ó Néill, ‘Dementia and Patient Safety: A Gewirthian Analysis’ (2019) 19(2–3) Medical Law International 182–206.

82 Gerards (n 50) 247.

83 E Örücü, ‘The Core of Rights and Freedoms: the Limit of Limits’ in T Campbell and others (eds), *Human Rights. From Rhetoric to Reality* (Blackwell 1986) 37–59 at 37.

84 G Van der Schyff, ‘Cutting to the Core of Conflicting Rights: The Question of Inalienable Cores in Comparative Perspective’ in E Brems (ed), *Conflicts Between Fundamental Rights* (Intersentia, 2008) 131–47 at 140, as cited in Gerards (n 50) 255.

85 See *Observer and Guardian v the United Kingdom* judgment of 26 November 1991, A216, para 72.

86
*Lingens* judgment of 8 July 1986, A 103, para 43.

87 S Greer, ‘The Exceptions to Articles 8 to 11 of the European Convention on Human Rights’ (Council of Europe Publishing 1997) <https://www.echr.coe.int/LibraryDocs/DG2/HRFILES/DG2-EN-HRFILES-15(1997).pdf> accessed 11 March 2022.

88 TL Beauchamp and JF Childress, *Principles of Biomedical Ethics* (7th edn, OUP 2013). Also see Katie Page, ‘The Four Principles: Can They Be Measured and to They Predict Ethical Decision Making?’ (2012) 13 BMC Med Ethics 10.

89 T Beauchamp, ‘The “four principles” Approach to Health Care Ethics’ in RE Ashcroft and others (eds), *Principles of**Health**Care**Ethics* (2nd edn, John Wiley & Sons Ltd 2007) 7. Keeling and Bellefleur consider these four principles in the context of the application of ethics in public health, as exemplified in a number of public health frameworks. See M Keeling and O Bellefleur, ‘“Principlism” and Frameworks in Public Health Ethics’ (National Collaborating Centre for Healthy Public Policy 2016) <http://www.ncchpp.ca/docs/2016_Ethics_Principlism_En.pdf> accessed 11 March 2021.

90 JAK Kegley, ‘Community, Autonomy, and Managed Care’ in G Mc Gee, *Pragmatic Bioethics* (2nd edn, MIT Press 2003) 240.

91 E Jackson, *Medical Law: Texts, Cases and Materials* (5th edn, OUP 2019) 17–18.

92 Kegley (n 90) 244.

93 See Sean McAleer, *Plato’s Republic: An Introduction* (Open Books Publishers 2020).

94
*BP v Surrey County Council and RP* [2020] EWCOP 17; *BP v Surrey County Council* [2020] EWCOP 22.

95 [2020] EWCOP 22.

96 REG Upshur, ‘Principles for the Justification of Public Health Intervention’ (2002) 93(2) Canadian Journal of Public Health 101–03.

## Notes

I wish to acknowledge the very helpful comments received from Charles Foster in relation to an earlier draft of this article. I am also grateful to the anonymous reviewers who provided extremely beneficial feedback. Engaging discourse and incisive debate following a presentation of some of the arguments in this article at the Society of Legal Conference Annual Meeting at Durham University 2021 helped to sharpen the focus of the narrative.

**Declarations:** no ethics approval required.

